# Insignificant effects of loss of heterozygosity in HLA in the efficacy of immune checkpoint blockade treatment

**DOI:** 10.1007/s13258-021-01207-8

**Published:** 2022-02-02

**Authors:** Yohan Yang, Eunyoung Kim, Sangwoo Kim

**Affiliations:** 1 Department of Biomedical Systems Informatics, Yonsei University College of Medicine, 50-1 Yonsei-ro, Seodaemun-gu, Seoul, 03722 South Korea; 2 Department of Biomedical Systems Informatics, Graduate School of Medical Science, Brain Korea 21 Project, Yonsei University College of Medicine, 50-1 Yonsei-ro, Seodaemun-gu, Seoul, 03722 South Korea

**Keywords:** Immunotherapy, Immune checkpoint blockade, Loss of heterozygosity in HLA, Tumor microenvironment

## Abstract

**Background:**

It is assumed that loss of heterozygosity and allelic copy loss in HLA gene is associated with poor response rates in immune checkpoint inhibitor treatment. H-owever, the accurate extents or consistency in cancer types have not been explored.

**Objective:**

The goal of this study is to investigate quantitative relationship between HLA allelic copy loss and response rates to immune checkpoint inhibitors. Also, tumor microenvironment was computationally assessed in the tumors with HLA copy loss to provide potential mechanisms for the relationships.

**Method:**

A total of 282 whole exome sequencing data from three cohorts of patients who received immune checkpoint blockade immunotherapy were analyzed, including Anti-PDL1 treated in metastatic urothelial cancer (N = 216), anti-PD1 treated metastatic melanoma (N = 26), and anti-CTLA4 treated metastatic melanoma (N = 39). The LOHHLA algorithm was used to calculate allelic copy number loss at each HLA-A, -B, and -C locus, and further determine HLA allelic copy loss status. The HLA copy status and ICB response rates were analyzed for association using Fisher’s exact test. The CIBERSORT-absolute algorithm was then used to analyze the patient's immune environment, which represented loss of heterozygosity, using paired matched RNA sequencing data.

**Results:**

Unlike the general expectation, HLA allelic copy loss was not significantly associated with the ICB responses. Moreover, the relationship showed a reversed relationship in HLA-A in the urothelial cancer (better ICB response in HLA copy loss). Regardless of the HLA copy status, the proportion of cytotoxic immune cells in the immune environment of patients was correlated with ICB response, which was higher in the loss of heterozygosity group in the urothelial cohort.

**Conclusion:**

Although the loss of heterozygosity in HLA was generally expected to be an inhibitory factor in the immune treatment response by causing T cell immune evasion, our analysis demonstrates no explicit relationships.

**Supplementary Information:**

The online version contains supplementary material available at 10.1007/s13258-021-01207-8.

## Introduction

Cancer consistently attempts to evade human immune system. So far, several factors that cause immune evasion have been identified including immune regulatory cells (Yokokawa et al. 2008), immunosuppressive mediators (Chen et al. 1994; Pasche 2001), and defective antigen presentation pathways (Maeurer et al. 1996). These mechanisms for immune evasion strongly affect the efficacy and response rates for cancer immunotherapy. Therefore, revealing and suppressing immune evasion factors is one of the most prioritized strategies to improve the response rate of cancer immunotherapy (Rosenberg 2014).

Among diverse treatment options, the immune checkpoint blockade (ICB) is of a particular interest, due to the unprecedented strong efficacy on previous refractory cancers such as metastatic melanoma (Hugo et al. 2016; Snyder et al. 2014) and lung cancer (Lynch et al. 2012; Rizvi et al. 2015). ICBs are typically monoclonal antibodies that target immune suppressive molecules in the cytotoxic T cell (CD-8) related immunity; these molecules include Programmed death protein1 (PD1), Programmed death-ligand1 (PD-L1) and cytotoxic T-lymphocyte-associated protein 4 (CTLA4) (Callahan et al. 2016).

Despite the potential responses with long overall survival, but response rate still remains limited (less than 20%) (Franklin et al. 2017). Several factors are closely related to the response of ICBs including immune phenotype, target gene expression and tumor mutation burden (Gaffney et al. 2019; Gao et al. 2016; Zaretsky et al. 2016). Another important factor is loss of functions in the antigen processing and presentation, which governs generation, recognition, binding and presentation to the cell surface. In particular, the loss of binding ability to the major histocompatibility complex (MHC) in the endoplasmic reticulum (ER) is a key mechanism for immune evasion in cancer, which is usually acquired by the loss of Human Leukocyte Antigen (HLA) gene that forms MHC protein (Chowell et al. 2018). HLA are highly polymorphic and determines the peptides to be bound and transported to the cell surface for T cell recognition (Hoof et al. 2009; Marty et al. 2017). Therefore, loss of HLA is expected to confer immune evasion. Similarly, loss of a copy (loss of heterozygosity, LOH) in HLA also leads to reduction in the neoantigens processing, due to the decrease of peptide coverages that can be recognized (Chowell et al. 2019). While LOH in HLA has been reported as one of the immune evasive mechanisms (Dejima et al. 2021; McGranahan et al. 2017), the exact effects and heterogeneity among cancer types have not been explored.

Here, we examined the clinical response of patients with loss of heterozygosity in 281 patients with ICB treatment and responses, in three different cancer types. We investigated the presence, frequency and patterns of LOH in HLA-A, B, and C genes and found diverse relationships between HLA LOH and ICB efficacy. Finally, we analyzed the tumor immune microenvironment along with the HLA LOH, to reveal the effects on immune cell compositions that may determine responses to ICBs.

## Materials and methods

### Dataset collection for ICB-treated patient cohorts and response evaluation

We used three data sets from the ICB trial to conduct the analysis. Anti-PDL1 treatment in metastatic urothelial cancer (EGAD0001003977; Mariathasan et al. 2018), anti-PD1 treatment in metastatic melanoma (GSE78220; Hugo et al. 2016) and anti-CTLA4 treatment in metastatic melanoma (phs000452.v2; Berger et al. 2012) are the three datasets that were matched with normal DNA, tumor DNA, and tumor RNA. Based on RECIST version 1.1 guideline (Lawrence et al. 2016), we defined complete response (CR) and partial response (PR) as a responder group, and stable disease (SD) and progressive disease (PD) as a non-responder group. Patients who were not evaluable (NE) or who were duplicated were excluded. As a result, we used 216 patients in EGAD0001003977, 26 patients in GSE78220, and 39 patients in phs000452.v2 to conduct the analysis.

### Data processing

We used the bwa-mem (v0.7.10) algorithm to align whole exome data to the human reference genome (hg38). The GATK (4.0.9.0) package was used to mark and fix duplicate reads in the alignment data.

We aligned RNA sequence data to the human reference genome (hg38) and gene transfer format in evidence-based annotation of the human genome (hg38), version 37 (Ensembl 103), using the STAR (v2.7.3a; Dobin et al. 2013) algorithm with 2-pass method.

### Gene expression quantification

Using an RSEM (Li et al. 2011) algorithm, gene expression was calculated using aligned RNA data. Transcripts per million (TPM) value was used in the analysis, and it was completed. The analysis was carried out within the cohort by dividing it into expression-high and expression-low groups based on the median of each gene.

### Genotyping and LOH status determination of HLA genes

Because 8-digit HLA genotypes are required to evaluate LOHHLA, we used the polysolver (Shukla et al. 2015) algorithm to genotype HLA in aligned normal DNA data. The LOHHLA (McGranahan et al. 2017) algorithm was used to assess loss of heterogeneity in HLA. The algorithm was used to determine whether the loss of the allele specific copy number was significantly different between the normal DNA data and the tumor DNA data. When the p-value was less than 0.01, it was determined that HLA allele loss had occurred in the corresponding HLA allele using the student t-test.

### Assessment of tumor immune microenvironment

The analysis was carried out using a CIBERSORT-absolute (Chen et al. 2018) algorithm that can perform immune cell profiling in order to identify the immune cells that compose the patient's tumor microenvironment. Using the expression data, we conducted an analysis for each cohort using the TPM of each gene.

### Statistical methods

Fisher's exact test was used to assess the relationship between loss of heterozygosity in each HLA and clinical response within the cohort. It was determined that there was a significant difference between the two groups if the p value was less than 0.05.

The Wilcoxon rank-sum test was used to determine whether the difference between the two groups of estimated immune cell score was significant when comparing allelic copy number loss and allelic copy number intact groups. It was determined that there was a significant correlation between the two groups if the p-value was less than 0.05.

Log-rank test was used to calculate the difference in survival rates between the two groups in terms of the patient's overall survival. It was determined that there was a significant correlation between the two groups if the p value was less than 0.05.

## Results

### Identification of HLA genotypes and allelic copy loss

**Fig. 1:**
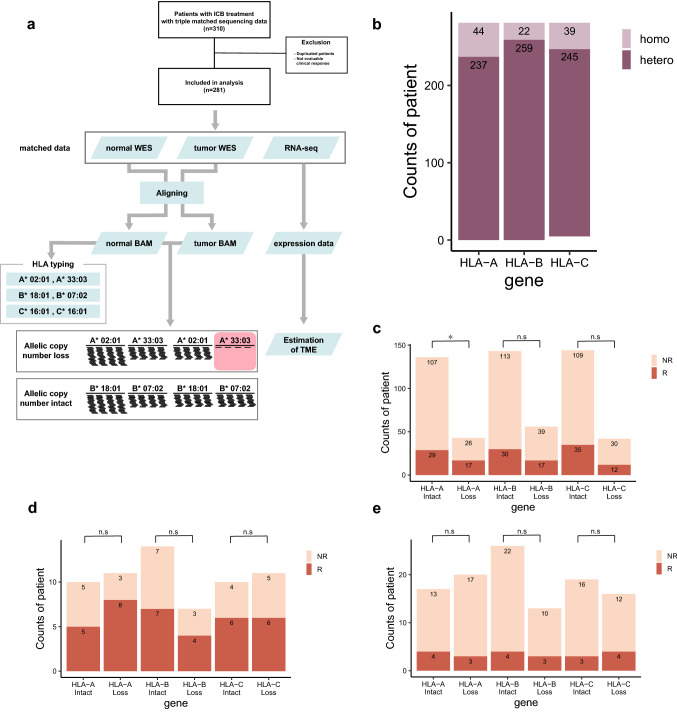
Patients who received ICB treatment and their overall HLA status for each cohort. (**a**) Examine each patient's workflow. (**b**) Total HLA genotype in three cohorts. The number of patients with allelic copy number loss and intact in each HLA gene, as well as clinical response information, are shown in (**c**) metastatic urothelial cancer with anti-PDL1, (**d**) metastatic melanoma with anti-PD1 and (**e**) metastatic melanoma with anti-CTLA4. The asterisk indicates that the p-value (Fisher's exact test) was less than 0.05

Genotyping of three HLA genes (HLA-A, -B, and -C) was attempted to determine allelic loss from the 281 patients based on whole-exome sequencing data (Fig. 1a, see Methods). Initially, HLA zygosity was predicted to remove homozygous HLA genes; allelic loss cannot be identified in homozygous HLA genes. After homozygosity removal, 237, 259, and 245 heterozygous HLA-A, -B, and -C were used for further analysis (Fig. 1b). Analysis of loss of heterozygosity (see Methods) identified allelic loss in three HLA genes, and grouped all patients into (a) HLA allelic copy intact and (b) HLA allelic copy loss groups. We found that 74 (31.2%), 76 (29.3%), and 69 (28.2%) of HLA-A, -B, and -C genes had allelic copy loss, respectively.

### Association between HLA allelic copy loss and response to ICBs

We investigated if the HLA allelic copy loss is associated with response rates to ICB treatment. Previous analysis and theoretical assumptions lie in the negative response in HLA allelic copy loss groups; the reduction in the MHC binding would lead to decreased T cell immunity, followed by weak ICB efficacy. To test the relationship between HLA allelic copy loss and ICB treatment response, we used pre-annotated clinical information (NR: non-response, and R: response; Table S1) and their HLA compositions for grouped comparisons (Fisher’s exact test).

Statistical analysis in the metastatic urothelial cohort (N = 216) revealed unexpected relationships between the two groups. Unlike the expectation, HLA allelic copy loss was not associated with ICB responses in HLA-B and -C genes (Fig. 1c). Moreover, we found an reversed association in HLA-A; the HLA-A allelic loss was enriched in the good response group (Fig. 1c). This unexpected tendency also appeared in the same analysis with respect to overall survival (Fig S1). We found that the non- or reversed association was reproduced in two other cohorts (metastatic melanoma with anti-CTLA4 treatment, N = 26 and with anti-PD1 treatment, N = 39) (Fig. 1d and 1e), confirming the weak or no relationship of HLA allelic copy number states and ICB response.

### Tumor immune microenvironment analysis according to HLA allelic copy loss status

**Fig. 2:**
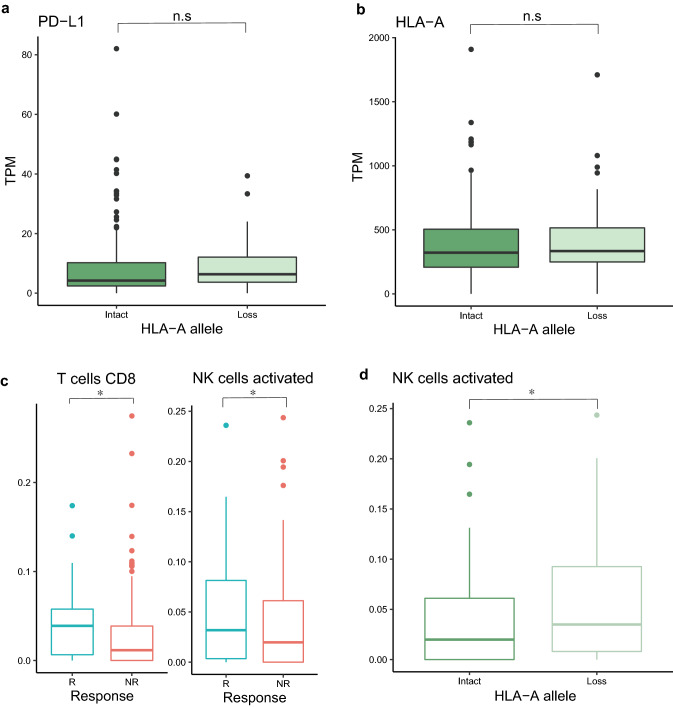
Immune-related gene expression and immune profiling score in metastatic urothelial cancer. (**a**) Differences in the expression of the ICB target gene (PD-L1) between the allelic copy number intact and allelic copy number loss groups. (**b**) Differences in the expression of HLA-A between the allelic copy number intact and allelic copy number loss groups. (**c**) The difference in cytotoxic immune cell profiling scores between responders and non-responders. (**d**). The difference in NK cell immune profiling scores between allelic copy number intact and allelic copy number loss groups. The asterisk indicates that the p-value (Wilcoxon rank-sum test) was less than 0.05

We identified tumor microenvironment in cohorts with ICB treatments to better understand immune related molecule components when a patient had loss of heterozygosity in HLA. Clinical response was related to the expression of ICB-targeted genes. Furthermore, we hypothesized that HLA allelic loss was correlated with decreased HLA expression. As a result of the significant relationship between responder and HLA-A loss of heterozygosity, we expected that the expression of the targeted gene was enriched while the expression of the HLA gene was decreased. We also observed that genes that are targeted by ICBs were associated with overall survival in an expression-high group at each cohort (Fig S2). However, there were no significant differences in the expression of immune-related genes (Fig. 2a and Fig. S3). In HLA-B, and -C, the differences in expressions of immune-related genes were not statistically significant between other allelic loss groups and allelic intact groups. (Fig. 2b and Figs. S3, 4, 5). The differences in expressions of immune-related genes were statistically significant in anti-PD1 treatment data with LOH in HLA-B but that was not related overall survival with ICB-target gene expression-high group (Figs. S2 and S4). These findings show that HLA allelic loss in DNA has no explicit effect on HLA gene expression or immune gene expression.

Next, we performed computational immune cell profiling analysis on each patient to identify immune cell compositions in the presence of loss of heterogeneity in HLA. The analysis confirmed that among the immune cell scores, the cytotoxic immune cell score was significantly higher in patients with clinical response, regardless of the HLA loss of heterozygosity (Fig. 2c), which is consistent with the previous reports (Mariathasan et al. 2018). Therefore, it appears to be beneficial to use ICB treatment if cytotoxic immune cells are present in a large volume in the inspected tumors. On the other hand, we confirmed that only NK cells among the cytotoxic immune cell scores had a higher score in the allelic copy number loss in HLA-A group when comparing the immune cell score of patients in the allelic copy number loss group with patients in the allelic copy number intact group (Fig. 2d). These results show that cytotoxic immune cells do not show significant differences in the allelic copy number loss group, but NK cells appearing in the tumor microenvironment of the patients appear more in the allelic copy number loss group.

## Discussion

We conducted this study to determine whether HLA allelic loss in patients with ICB treatment was associated with the patient's clinical response. As a result, we chose three ICB-treated cohorts and used whole exome sequencing to try to find the allelic copy number loss in HLA. Because detection of the cytotoxic immune cell was difficult when patients had loss of heterozygosity in HLA, cancer was not removed. As a result, even if ICB treatment improves the tumor microenvironment, it was not thought to be beneficial for clinical response. However, our findings revealed that HLA-A allelic loss did not differ significantly between responders and non-responders, and that HLA-A allelic loss was more beneficial for clinical response in metastatic urothelial cancer. As a result of this finding, it was determined that loss of heterozygosity could not be used as a predictor of ICB treatment response.

If loss of heterozygosity does not show disadvantages in the immune environment, immune cell profiling was conducted to determine what immune environment it is composed of. The loss of heterozygosity in the HLA group was expected to have little effect on NK cells and cytotoxic T cells, which are directly affected by HLA. As a result, in metastatic urothelial cancer, cytotoxic immune cells were found in higher numbers in the responder group. Following that, we analyzed how immune cells correlated with loss of heterozygosity in HLA, and found that patients with loss of heterozygosity in HLA-A had more NK cells. From this perspective, loss of heterozygosity in HLA is not expected to be a factor that reduces immune therapy response, but rather results in a later reaction as a result of the immune evolutionary mechanism. Due to immune pressure, cancer evolved in a way that does not present neoantigen, and because an immunogenic environment has already been created, it is thought that cancer would be more suitable for clinical response when the immune inhibitor's function is limited by ICB.

Loss of heterozygosity in HLA has been thought to be an important marker for predicting ICB treatment response, but this analysis shows that it is both significant and unrelated. Instead, loss of heterozygosity in HLA acts as an immune evolutionary factor in the immunogenic tumor microenvironment, so some cancer types benefit from ICB treatment. Because cancer is caused by a complex set of factors, future research should take into account not only immune factors but also other factors when assessing cancer and deciding how to proceed with treatment.

## Supplementary Information

Below is the link to the electronic supplementary material.

Supplementary file1 (XLSX 32 KB)

Supplementary file2 (EPS 1472 KB)

Supplementary file3 (EPS 1427 KB)

Supplementary file4 (EPS 1508 KB)

Supplementary file5 (EPS 1628 KB)

Supplementary file6 (EPS 1617 KB)
